# Acute effects of angler’s groundbaits: nutrient flux to water column

**DOI:** 10.1038/s41598-023-44381-3

**Published:** 2023-10-17

**Authors:** Dorottya Lilla Fazekas, László Antal, Béla Halasi-Kovács, Maciej Kwiatkowski, Flórián Tóth, András Specziár, Attila Mozsár

**Affiliations:** 1 Research Centre for Fisheries and Aquaculture, Institute of Aquaculture and Environmental Safety, Hungarian University of Agriculture and Life Sciences, Szarvas, 5540 Hungary; 2 Pál Juhász-Nagy Doctoral School of Biology and Environmental Sciences, University of Debrecen, Debrecen, 4032 Hungary; 3 Department of Hydrobiology, University of Debrecen, Debrecen, 4032 Hungary; 4 Hel Marine Station, University of Gdańsk, 84-150 Hel, Poland; 5 HUN-REN Balaton Limnological Research Institute, Tihany, 8237 Hungary; 6 National Laboratory for Water Science and Water Security, HUN-REN Balaton Limnological Research Institute, Tihany, 8237 Hungary

**Keywords:** Limnology, Environmental impact, Freshwater ecology

## Abstract

Although ground-baiting related nutrient loading has been widely studied, we do not know what proportion of these nutrients release into the water column, affecting primary production directly. We conducted short-term (24-h, 5-day) experiments at wide temperature range, in presence and absence of fish using fish meal-based (FM-GB) and plant-based groundbait (PB-GB), to assess the nitrogen (N) and phosphorus (P) fluxes from GB into the water column. Nitrogen release from unconsumed FM-GB was negligible in the first 3 days, then increased abruptly, releasing 32% of its total N content by the fifth day. In contrast, PB-GB acted as temporary sink for inorganic N forms. Considerable (18–21%) inorganic P release was observed in both GB types in the first twelve hours. Consumed GBs induced considerable inorganic N release and its rate increased with temperature. Particulate forms predominated the released N in PB-GB, suggesting impaired digestion. Phosphorus—dominated by particulate forms—release was similar or lower than in unconsumed GB. Based on our results, excessive use of GB—when high amount of it remains unconsumed—can enhance eutrophication in P-limited ecosystems. Although less digestible GBs may have less abrupt effect on the primary production, undigested nutrients remain unavailable for removal through fish harvest.

## Introduction

In the last decades, human-induced external nutrient loadings highly accelerate eutrophication—referred as anthropogenic eutrophication—, making it a foremost water quality issue worldwide. Although recreational activities are especially sensitive to negative impacts of eutrophication^1,2^, they can also be important sources of both external and internal nutrient loadings in freshwater ecosystems^3,4^. Recreational fishing, especially angling—using hook, line, and rod for capturing fish^5^—is one of the most popular water-based outdoor activity; it concerns approximately 10% of population of industrialized countries^6,7^ and has substantial economic and socio-cultural importance^8^. To maximise the catch, groundbaits (hereafter, GB) are frequently used (i.e. ground-baiting) to attract fish (primarily cyprinids) to the fishing area^9–11^. More precisely, ground-baiting means that angler disperses GBs before and during fishing at the fishing spot to attract to and keep the fish around the baited hook. Mean daily bait input per angler generally varies between 1 and 2.3 kg in European freshwaters^9,12,13^. Due to the high and increasing number of anglers, the total amount of GB input to freshwaters can reach thousands of tons annually per country^10^ or even in a single lake^13^. Excessive use of GBs—due to lack of daily limits or high number of anglers in many areas—can cause nutrient enrichment and contribute to the anthropogenic eutrophication^14,15^.

Nutrient content of GBs and angler’s baiting habits (type and amount of GB used) were widely studied to assess the extent of ground-baiting related nutrient loading (e.g.^9,16,17^), its contribution to anthropogenic eutrophication and to elaborate adaptive management strategies^13,14^. Reliable risk assessment of ground-baiting, however, also requires knowledge of nutrient fluxes from GBs and their drivers. In short-term, nutrients from GB introduced into the water can flux into three pools: (i) sequester in fish body, (ii) sediment in benthos and (iii) dissolve or suspend (fine faces and GB debris) in water column. Fish body and sediment, at least temporarily—the turnover rate depends on the life span of fish, sediment characteristics and sedimentation rate—, represent nutrient sinks for water column. Although nutrient flux from these pools into the water column is obvious at wide time-scale, permanent nutrient loss also occurs (e.g. incomplete decomposition of fish carcass, see^18^; and sedimentation, see^19,20^). In contrast, dissolved nutrient release into the water column directly affects the pelagic primary producers. Actual contribution of ground-baiting to anthropogenic eutrophication, therefore, highly depends on the amount and form of nutrients fluxed into the water column.

Partitioning of nutrients among fish (and other consumers, e.g. macroinvertebrates) body, sediment and water column is a function of solubility and digestibility properties of GB. Well-soluble nitrogen and phosphorus forms are rapidly dissolved into the water column. In unconsumed GB, dissolution is the only path of nutrient flux from GB to the water column. Nutrients of consumed GB are potentially sequestered in fish body, but it is a function of digestibility^21,22^. Sequestration increases with increasing digestibility but coupled with high nutrient content, it can result in dissolved nutrient release in water column, because fish release the nutrients beyond their demand^23^. Insoluble and undigested or indigestible parts (GB, faces and their coarse debris) sink to the bottom and their nutrients can be temporally trapped in sediment (benthos).

Composition of GB products currently available in market differs considerably: being composed purely of animal- (e.g. fish-, meat- and mussel meal) or plant-derived ingredients (e.g. ground cereals) and consisted of their mixture. Solubility of ingredients used in GB manufacturing are unknown; sporadic data are available only for aquaculture fish feeds^24–26^. On the contrary, considerable differences in digestibility between animal- and plant-derived ingredients are well known^27^. Fish meal and most of the animal-derived ingredients are highly available for fish^28^, but deficiencies in essential amino acids and presence of antinutritional factors in plant-derived ingredients lead to impaired digestion^29^.

In this study, we examined the ground-baiting related short-term nutrient flux into the water column. Specifically, (i) we revealed the nitrogen (N) and phosphorus (P) release from unconsumed and consumed fish meal- and plant-based GBs by conducting short-term laboratory and outdoor tank experiments in the presence and absence of fish. Further, because the temperature accelerates both dissolution and digestion efficiency of fish^24,30^, (ii) we also evaluated the influence of temperature on nutrient release.

## Results

### Nutrient content of GBs

Both N and P content was higher for FM-GB. The N content, expressed as percentage of wet mass and dry mass in parentheses, was 6.01% (6.37%) for FM-GB and 4.01% (4.81%) for PB-GB. The P content was 1.04% (1.10%) for FM-GB and 0.84% (1.01%) for PB-GB.

### Nutrient dissolution from GBs

**Figure 1:**
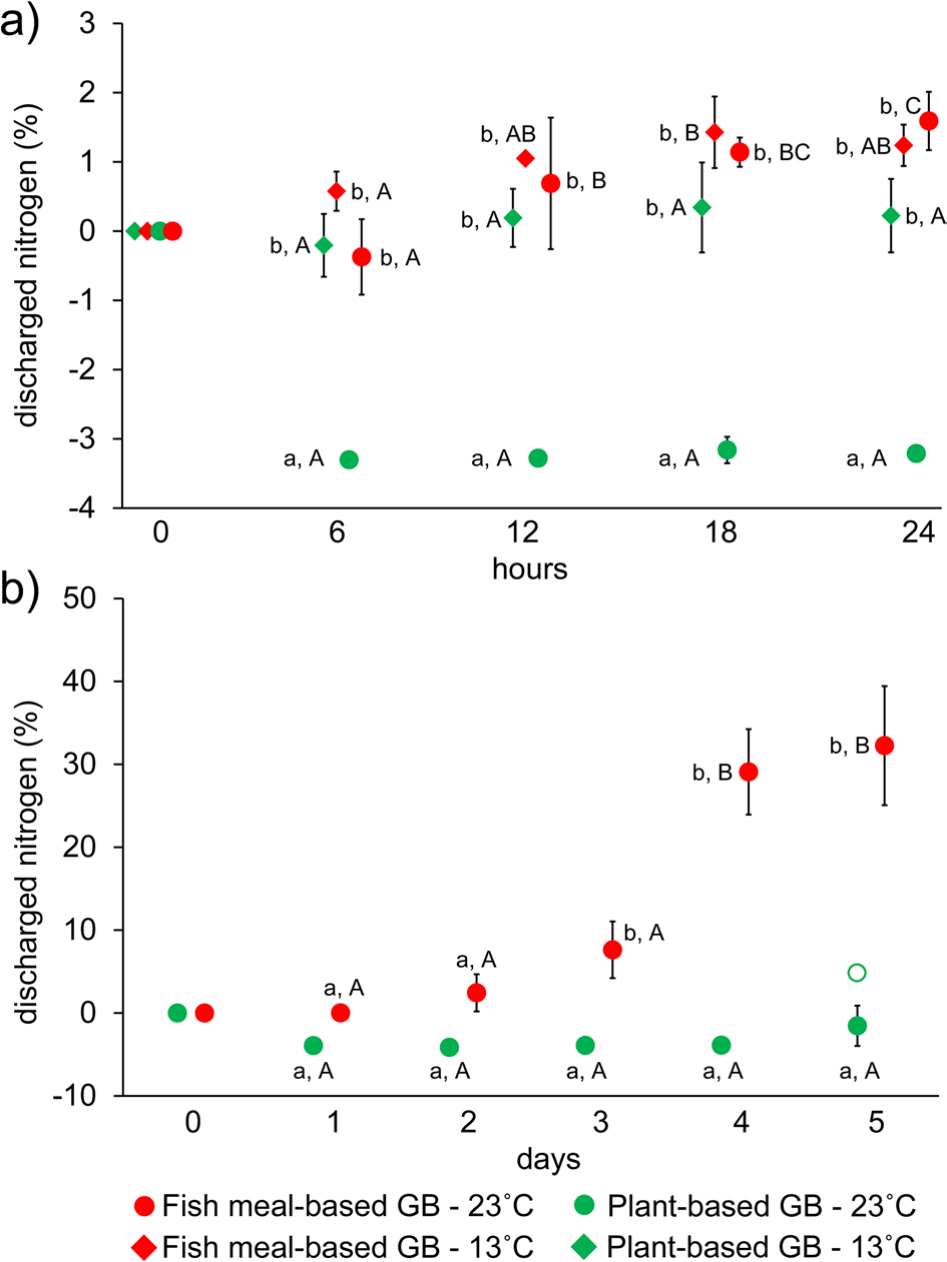
Nitrogen dissolution from groundbaits (GB) at 13 °C and 23 °C during the first 24 h (**a**) and at 23 °C during the first 5 days (**b**). Plotted values not sharing any common latter are statistically different at Bonferroni adjusted p < 0.05 level at a given time point (lower case letters; between GB type × temperature treatment differences) or in time (upper case letters; within treatment differences). Empty green circle denotes outlier data. To improve the readability of the graph, the data are shown slightly offset to either side of the relevant x-axis value.

Analysis of N dissolution from GB to tap-water data of the 24-h long experiment revealed a significant three-way interaction between the time, temperature and GB type, and for each level of time, significant two-way interaction was found between temperature and GB type (Table 1). Specifically, at 13 °C, dissolution of N was very limited (< 2%) from both GBs and with slight (FM-GB) or no (PB-GB) statistically significant variation in time (Fig. 1a). At 23 °C, dissolution of N was also low (< 2%) from FM-GB, but increased slightly in time. Conversely, PB-GB showed a stable (i.e. no variation in time) negative N dissolution value (i.e. the inorganic N content of the tank water decreased) averaging between − 1.5 and − 4.1% of the initial N content of the introduced GB (Fig. 1a). Analysis of the 5-day long experiment at 23 °C also revealed a significant interaction between time and GB type (Table 2). In the FM-GB, dissolution of N started to increase rapidly after day three and its average reached 32% at day five (Fig. 1b). While, PB-GB again showed a stable negative N dissolution value during the 5-day long observation period.

**Table 1:** Result of mixed-design ANOVA testing the effects of groundbait (GB) type and temperature in time on nitrogen (N) and phosphorous (P) dissolution during 1-day long laboratory experiment.

Parameter	Terms	SS	df	MS	F	p
N%	Intercept	4.80	1	4.80	8.51	0.019
	Temperature	40.78	1	40.78	72.36	< 0.001
	GB type	73.09	1	73.10	129.71	< 0.001
	Temperature × GB type	28.18	1	28.18	50.00	< 0.001
	Error	4.51	8	0.56		
	Time	4.81	3	1.60	25.41	< 0.001
	Time × temperature	0.39	3	0.13	2.08	0.129
	Time × GB type	1.87	3	0.62	9.88	< 0.001
	Time × temperature × GB type	1.04	3	0.35	5.50	0.005
	Error	1.51	24	0.06		
P%	Intercept	10,883.54	1	10,883.54	805.33	< 0.001
	Temperature	169.88	1	169.88	12.57	0.007
	GB type	343.83	1	343.83	25.44	< 0.001
	Temperature × GB type	1.31	1	1.31	0.10	0.763
	Error	108.11	8	13.51		
	Time	1885.32	3	628.44	341.14	< 0.001
	Time × temperature	11.11	3	3.70	2.01	0.139
	Time × GB type	43.63	3	14.54	7.89	< 0.001
	Time × temperature × GB type	1.24	3	0.41	0.22	0.878
	Error	44.21	24	1.84

**Table 2:** Result of mixed-design ANOVA testing the effect of groundbait (GB) type in time on nitrogen (N) and phosphorous (P) dissolution during 5-day long laboratory experiment.

Parameter	Terms	SS	df	MS	F	p
N%	Intercept	966.57	1	966.57	54.87	0.002
	GB type	2222.97	1	2222.97	126.19	< 0.001
	Error	70.46	4	17.62		
	Time	1713.78	4	428.45	40.00	< 0.001
	Time × GB type	1141.57	4	285.39	26.64	< 0.001
	Error	171.38	16	10.71		
P%	Intercept	14,453.29	1	14,453.29	1718.22	< 0.001
	GB type	7.51	1	7.51	0.89	0.398
	Error	33.65	4	8.41		
	Time	365.25	4	91.31	8.31	< 0.001
	Time × GB type	132.79	4	33.20	3.02	0.049
	Error	175.89	16	10.99		

**Figure 2:**
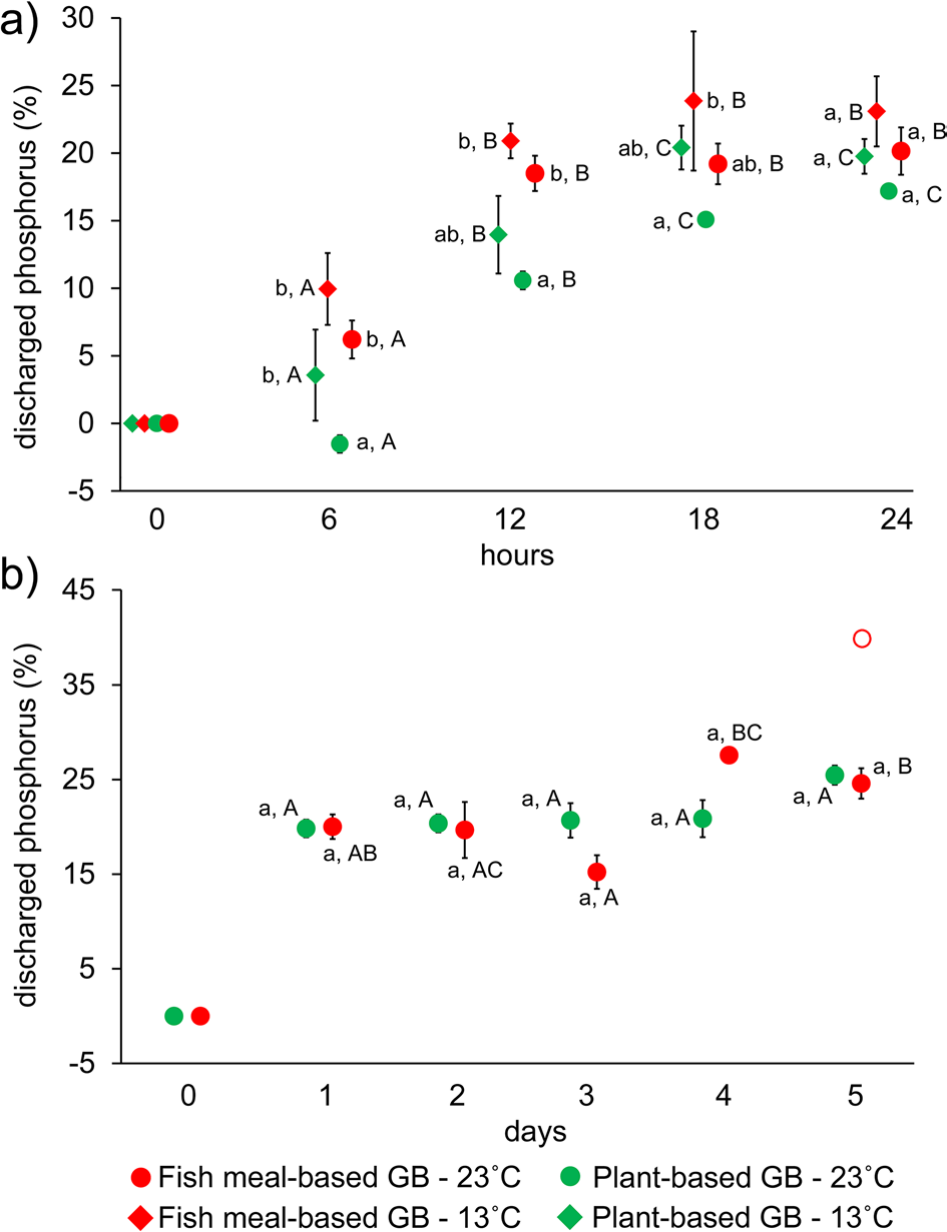
Phosphorous dissolution from groundbaits (GB) at 13 °C and 23 °C during the first 24 h (**a**) at 23 °C during the first 5 days (**b**). Plotted values not sharing any common latter are statistically different at Bonferroni adjusted p < 0.05 level at a given time point (lower case letters; between GB differences) or in time (upper case letters; within GB differences). Empty red circle denotes outlier data. To improve the readability of the graph, the data are shown slightly offset to either side of the relevant x-axis value.

Analysis of P dissolution from GB to tap-water data of the 24-h long experiment revealed a significant two-way interaction between time and GB type, and a simple main effect of temperature (Table 1). Significant main effect of GB type was approved for each level of time as well, while the effect of temperature proved to be non-significant based on the Bonferroni corrected p criteria. Specifically, at both 13 °C and 23 °C, dissolution of P increased during the first 12 and 18 h from the FM-GB and PB-GB, respectively, and plateaued in all four treatments (i.e. temperature × GB type) between 17 and 26% at 18–24 h after the addition of GBs (Fig. 2a). During the first 6 h, less percentage of P dissolved from the PB-GB at 23 °C, but this deviation diminished by the end of the 24-h long observation period. Analysis of the 5-day long experiment at 23 °C also revealed a significant interaction between time and GB type (Table 2). However, neither a consequent trend in time nor a difference between GB types at any level of time could be revealed by the post-hoc tests; dissolution of P values remained in the range of 14–28% (outlier: 40%; FB-GB, day 5) from day one to day five in both GB types (Fig. 2b).

### Nutrient release from GBs in presence and absence of fish

**Figure 3:**
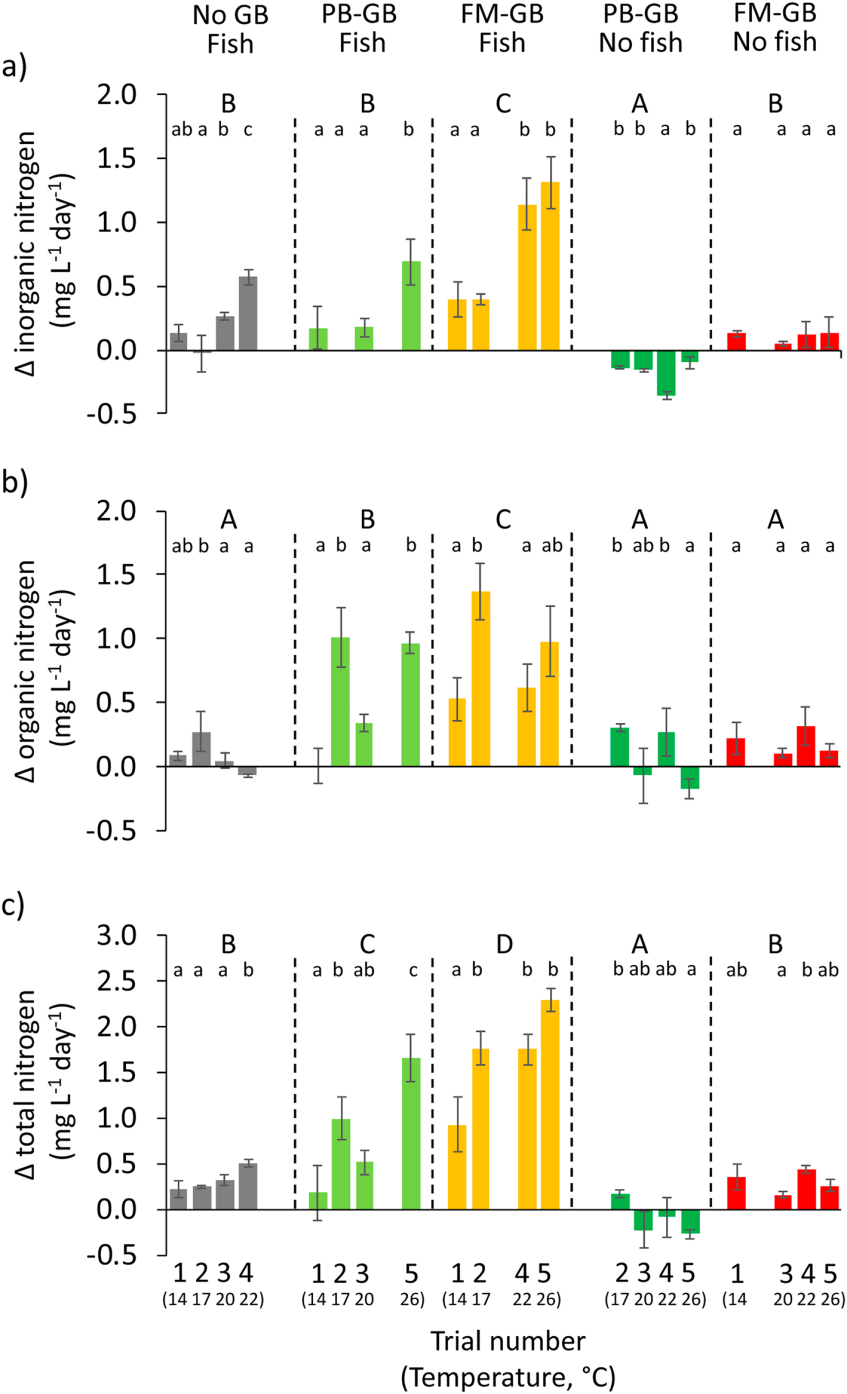
Changes of inorganic (**a**), organic (**b**) and total (**c**) nitrogen concentrations in experimental tank water depending on the type of the groundbait (plant-based ground-bait: PB-GB; fish meal-based ground-bait: FM-GB; No-GB) added and the presence or absence of fish in five consecutive 1-day long trials. Plotted values not sharing any common latter are statistically different at Bonferroni adjusted p < 0.05 level; lower case letters indicate within treatment (between trial) and upper case letters indicate between treatment differences. Note that water temperature varied between trials, and its values are indicated below the trial numbers in brackets. Note also that each treatment participated only in four of the five trials according to the balanced incomplete block design of the experiment.

**Figure 4:**
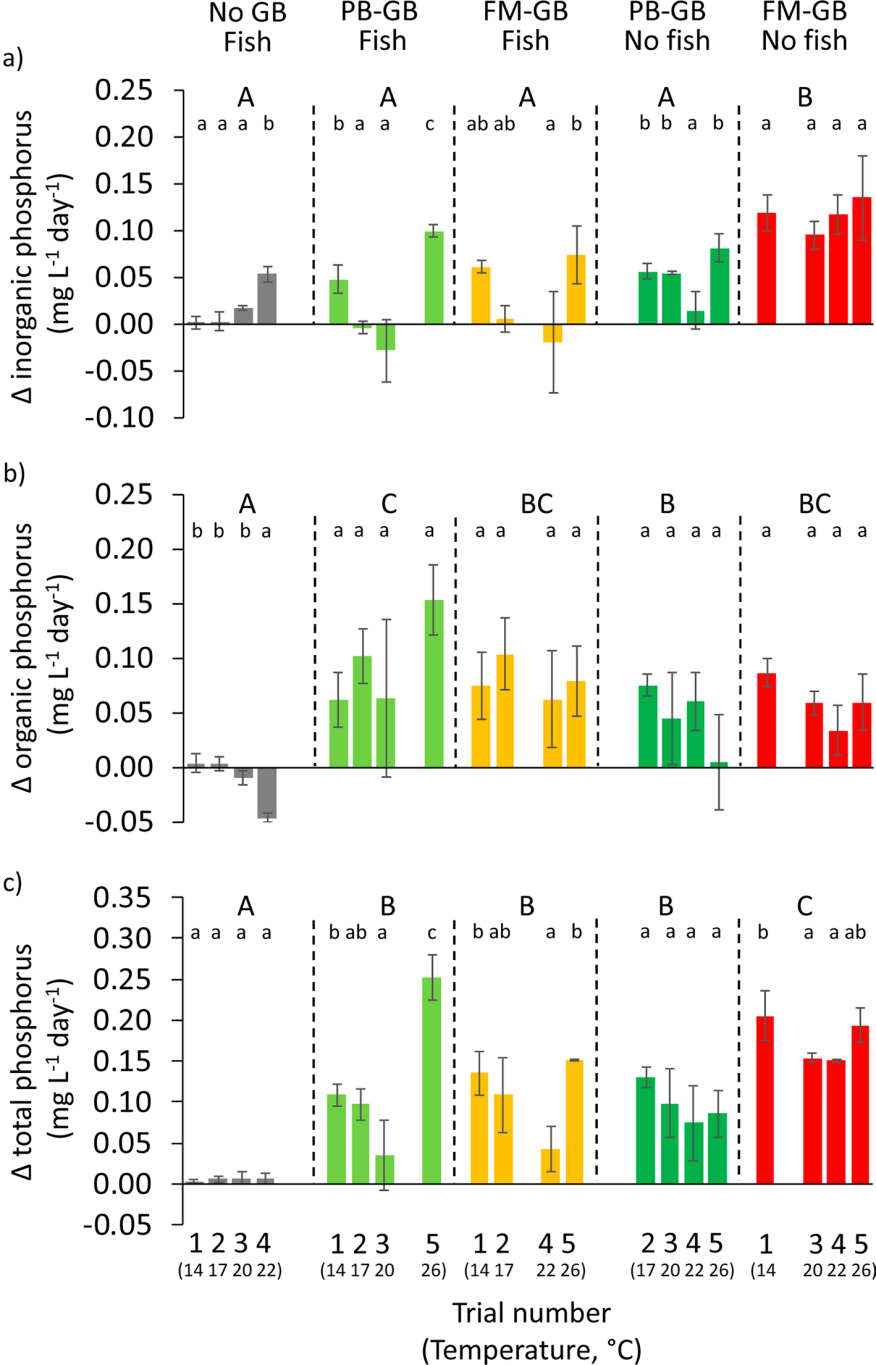
Changes of inorganic (**a**), organic (**b**) and total (**c**) phosphorous concentrations in experimental tank water depending on the type of the groundbait (plant-based ground-bait: PB-GB; fish meal-based ground-bait: FM-GB; No-GB) added and the presence or absence of fish in five consecutive 1-day long trials. Plotted values not sharing any common latter are statistically different at Bonferroni adjusted p < 0.05 level; lower case letters indicate within treatment (between trial) and upper case letters indicate between treatment differences. Note that water temperature varied between trials, and its values are indicated below the trial numbers in brackets. Note also that each treatment participated only in four of the five trials according to the balanced incomplete block design of the experiment.

In outdoor experiments, concentration changes (Δ values) of N and P forms differed between trials (expect in Δ organic P) and treatments (Figs. 3 and 4), but more variance was associated to treatment in each nutrient forms (Table 3). Between treatment differences were also significant within each trial (F_3,8_ = 6.45–187.17, p = 0.016–p < 0.001), except two (trial 5 for Δ inorganic P, and trial 3 for Δ organic P) of the thirty cases. Because temperature increased consistently and substantially between consecutive trials, monotonic between trial differences in Δ nutrient values by treatment types are considered as temperature-associated changes.

**Table 3:** Results of GLM analysis testing the effects of various groundbait (GB) type × fish treatments on nitrogen (N) and phosphorous (P) concentration changes in outdoor tank experiments with five, 1-day long trials.

Parameter	Terms		SS	df	MS	F	p	Model		
								F	p	R^2^_adj_
Δ Inorganic N	Intercept	Fixed	3.58	1	3.58	6.75	0.061	25.48	< 0.001	0.77
	Experimental round	Random	2	4	0.5	12.22	< 0.001			
	Treatment	Fixed	6.55	4	1.63	40.08	< 0.001			
	Error		2.08	51	0.04					
Δ Organic N	Intercept	Fixed	7.91	1	7.91	13.04	0.023	18.42	< 0.001	0.7
	Experimental round	Random	2.28	4	0.57	10.31	< 0.001			
	Treatment	Fixed	5.19	4	1.29	23.4	< 0.001			
	Error		2.82	51	0.05					
Δ Total N	Intercept	Fixed	22.15	1	22.15	24.35	0.008	34.98	< 0.001	0.82
	Experimental round	Random	3.43	4	0.85	9.18	< 0.001			
	Treatment	Fixed	21.52	4	5.38	57.58	< 0.001			
	Error		4.76	51	0.09					
Δ Inorganic P	Intercept	Fixed	0.14	1	0.14	16.57	0.016	14.47	< 0.001	0.65
	Experimental round	Random	0.03	4	0.01	8.73	< 0.001			
	Treatment	Fixed	0.06	4	0.01	16.69	< 0.001			
	Error		0.04	51	0.001					
Δ Organic P	Intercept	Fixed	0.17	1	0.17	77.84	0.001	8.86	< 0.001	0.52
	Experimental round	Random	0.01	4	0.002	1.68	0.168			
	Treatment	Fixed	0.07	4	0.01	13.96	< 0.001			
	Error		0.06	51	0.001					
Δ Total P	Intercept	Fixed	0.62	1	0.62	51.35	0.002	16.4	< 0.001	0.68
	Experimental round	Random	0.04	4	0.01	6.62	< 0.001			
	Treatment	Fixed	0.14	4	0.03	20.65	< 0.001			
	Error		0.08	51	0.001					

In fishless treatments, inorganic and total N and P concentrations increased significantly more in FM-GB than in PB-GB treatment, whereas Δ organic N and P values were similar in the two GB types (Figs. 3 and 4). Although between trials differences existed in some relations, neither of these effects were monotonic along the temperature range. Delta total N values ranged from 3.0 to 8.3% of the initial N content of the FM-GB, and inorganic N forms constituted 27.5 to 51.5% of the release. Similarly as in the laboratory experiments, in PB-GB, negative Δ inorganic N values were measured (Fig. 3a). Delta total P values ranged from 16.7 to 24.0% in FM-GB and from 10.2 to 17.1% in PB-GB of the initial P content of the GBs. The proportion of inorganic P within the total release varied considerably among trials and ranged between 58.0–77.4% in FM-GB and 18.9–94.1% in PB-GB.

In treatments including fish, GBs were rapidly consumed. Delta inorganic N and Δ total N tended to increase with temperature (Fig. 3a,c). Higher Δ N values were measured in FM-GB than in PB-GB and in No-GB treatments (Fig. 3). Delta total N constituted a similar proportion of initial N content of FM-GB (16.9–47.4%) and PB-GB (4.2–45.4%), but it related more to inorganic N in FM-GB. The Δ P values were significantly higher for treatments with GBs then in the presence of fish only. In turn, Δ P values showed no clear temperature dependency (i.e. monotonic changes on the course of trials) in any of the treatments with fish (Fig. 4) as well as no differences were found between the effect of PB-GB and FM-GB. Delta total P seemed to be more related to organic than to inorganic P forms in both GBs (Fig. 4), and its amount varied between 4.2–18.2% and 5.2–32.4% of the initial P content of FM-GB and PB-GB, respectively.

Delta N values were 2.3- to 9.4-fold higher in GB treatments in the presence than in the absence of fish (Fig. 3). However, on Δ P values, effect of fish was not uniform in GB treatments (Fig. 4); Δ inorganic and total P values were 3.8- and 1.6-fold higher in FM-GB treatments while Δ organic P values were 2.1-and 1.3-fold lower in PB-GB and FM-GB treatments in the absence than in the presence of fish.

## Discussion

Recognising the potential adverse effects of GB use, nutrient content of GBs has been investigated widely to assess ground-baiting related nutrient loading and net nutrient balance of recreational angling (e.g.^9,16,17^). Yet, there is scarce information on what proportion of nutrient introduced through GB use fluxes straight to the water column, directly enhancing the pelagic primary production. By conducting short-term laboratory and outdoor tank experiments in presence and absence of fish using fish meal- (FM-GB) and plant-based groundbaits (PB-GB), we demonstrated that a considerable part of nutrients stored in GB can be released (by fish for consumed GB) or dissolved (by dissolution for unconsumed GB) into the water column within a day. Our findings suggest the key role of GB manufacturing in reducing the effects of ground-baiting on nutrient dynamics of recipient ecosystems and pointed out the risks of excessive use of GB.

The rapid P release indicated the presence of water-extractable P forms in both FM-GB and PB-GB. Formulated fish feeds used in aquaculture are usually also rich in water-extractable P^24–26^ and show similarly rapid P release^26^. Nitrogen release requires the decomposition of N rich organic compounds^26^, and therefore, it is a delayed process in comparison with P release^24,25^. In FM-GB, considerable N release occurred after 3–4 days, which is typical also for animal-derived aquaculture feeds (c.f.^24,25^). On the contrary, PB-GB induced abrupt decrease of dissolved N. Although we used tap water, GB could serve as a vector for microbes. Inorganic N immobilisation, therefore, could be the result of microbial N uptake, which is common in the initial phase of litter (or other plant residues) decomposition^31,32^. A comparative study on decomposition of plant and animal residues documented similar difference in N immobilization^33^ as dissolved N demand of microbes increases with decreasing N:P molar ratio of substrate^34^. The decrease of inorganic N concentration was more emphasized in higher temperature, confirming the role of microbial activity in N immobilization. Based on our findings, unconsumed GB can constitute considerable source of orthophosphate, a directly available P-form to primary producers. Because European lakes are mostly P-limited^35^, direct contribution of unconsumed GB to anthropogenic eutrophication is highly assumable. Abrupt orthophosphate dissolution and dissolved N immobilization of plant-based GB decrease strongly the N:P ratio of available nutrients in water column and potentially favouring N-fixing cyanobacteria (c.f.^36^). Consequently, future researches could examine how excessive GB use—where the ratio of unconsumed GB is higher, e.g. angling competition—and different GB types alter primary production and phytoplankton composition.

Animals release nutrients both in particulate and dissolved forms. Undigested or indigestible compartments of ingested food are released in particulate forms (referred as egestion), while the digestible part can be assimilated by animals. To maintain a relatively constant body composition during the growth, animals retain the assimilated nutrients in a rate of their nutrient composition (ecological stoichiometry theory^37^), and nutrients in surplus are released in dissolved forms (referred as excretion). Beyond the nutrient content, therefore, the digestibility properties of food play also a crucial role in nutrient release^22^.

The majority of N in GB (and in food in general) are bound in amino acids, thus the amount and forms of released N is a function of protein content and digestibility^38^. The FM-GB had higher N and crude protein content and contained highly digestible animal-derived protein sources (e.g. fish meal, meat meal^28^). On the contrary, plant proteins of PB-GB might be less available for fish due to deficiencies in essential amino acids and the presence of antinutritional factors (e.g. non-starch polysaccharides, polyphenols, phytic acid and protease inhibitors) in plant ingredients^27^. Plant-based GB consumption, therefore, induced less and mostly particulate N release into the water column (c.f.^39^).

Although the P intake can be an important predictor of P release^22^; under natural conditions (i.e. food resources, P contents and rations) the requirement of fish usually exceeds the available P content of food which leads inconsistencies in P excretion (e.g.^40,41^). Total P content of GBs (1.04% in FM-GB and 0.84% in PB-GB) were slightly higher than the requirement of common carp (0.6–0.7% water-extractable P fed ad libitum;^42^) but their digestibility is doubtful. The fish- and bone meal contains high amount of tricalcium phosphate^43,44^ and many plant ingredients are rich in phytic acid^45^. Both are unavailable for monogastric species, like common carp, due to the absence of gastric juice and specific enzymes^46^. These indigestible P forms could contribute the dominance of particulate P in released material. Fish presumably retained the digestible P content of GBs, and the excretion remained close to the inevitable loss^47^ modelled by unfed fish.

The metabolism of poikilotherms highly depends on temperature (metabolic theory of ecology^48^), resulting in temperature-associated increase of nutrient excretion^23^, especially in N^49^ which is fuelled by protein oxidation^50^. By increasing enzyme activity, temperature affects positively the digestion efficiency for food in general and for many animal and plant proteins^30,51^. The fourfold dissolved N increment along the temperature gradient in our outdoor experiment was in accordance with previous studies: 10 °C temperature elevation induces 1.5 to 4-fold increase in N excretion^23,50^. This trend also occurred in unfed fish, since endogenous source of N excretion—in contrast to exogenous component, which is driven by N intake—is a function of temperature^52^. Although digestion and P assimilation efficiency increases with increasing temperature^30,53^, imbalances between available P content of GB and P requirements of common carps could kept the excretion rate close to the inevitable loss irrespectively from temperature.

Reducing the nutrient content of GBs is a reasonable measure to decrease the external nutrient loading through ground-baiting. Indeed, PB-GB contained less N than FM-GB, and PB-GB consumption resulted in less and mostly particulate N release to water column. The dominance of particulate N forms in released material indicates poor digestibility and low retention rate. Nutrient removal by fish harvest, however, is a keystone to counterbalance the nutrient loading and achieve neutral or even negative (i.e. net nutrient removal from the water) nutrient balance of recreational angling^13,14^. Although the indigestible ingredients reduce the dissolved nutrient release, and exerted less abrupt effect of phytoplankton productivity, but their nutrient content remains unavailable for removal through fish harvest, and thus accumulates and may represent longer-term environmental risk. The low nutrient content of GB alone, therefore, does not necessarily improve nutrient balance of recreational angling. Low and balanced (i.e. appropriate N:P molar ratio) nutrient content, slightly below the demand of fish combined with high digestibility can reduce the external nutrient loading (see also^21^) and ensure effective nutrient removal by catch-and-take fishing. Aquaculture feed production substantially improved the ecological footprint of fish nutrition in the last decades using novel, sustainable protein resources, avoiding indigestible ingredients (e.g. tricalcium phosphate) and increasing digestibility (e.g. heat- and enzyme treatment^28^). Goals of recreational angling and aquaculture highly overlap in fish feed production: both require highly attractive, nutritious feed and minimal waste. Establishing the achievements of aquaculture feed production in GB manufacturing can effectively move the recreational coarse angling towards sustainability. Any progress in improving nutrient retention in fish body, however, can be failed by prevailing volunteer catch-and-release practice (c.f.^13,14^). Therefore, complex angler education programs are also required to reach neutral nutrient balance of fisheries management.

Potential acute effects of ground-baiting are more pronounced during angling tournaments^13^, because of the excessive and highly concentrated use of GBs. Beyond the obviously increased external nutrient and organic matter loading, intensive ground-baiting increases also the proportion of unconsumed GBs. Our findings revealed that one-fifth of P content stored in GB can dissolved into the water column within 12 h. Abrupt increase of dissolved P in water column, however, directly affects the primary production and promote anthropogenic eutrophication. Strict bait-limit and use of low-P GBs, therefore, are more crucial during angling tournaments, especially in vulnerable ecosystems (i.e. long water retention, high shoreline-to-water surface area ratio and intensive angling pressure^13,14^). Decision-making at any stakeholder level and adaptive management strategies requires information about the nutrient content of GBs, which is currently unavailable for most of GB products. A stricter regulation of GB manufacturing, similarly to aquaculture feed production, should be in force to support fisheries management.

## Material and methods

### Experimental GBs

Because the most important difference in nutrient release was predicted between animal- and plant ingredients, we selected one fish meal- (hereafter FM-GB) and one plant-based formulated GB product (hereafter PB-GB). The FM-GB was moderately elongated pellet with 8 mm diameter and the PB-GB was ball-shaped boilie with 18 mm diameter. To ensure the similar surface-to-volume ratio and acceptability for fish, PB-GB was chopped into eight for experiments.

### Laboratory experiment—nutrient dissolution from GBs

To reveal purely dissolution-derived N and P release from FM-GB and PB-GB into the water and its temperature dependence, laboratory experiments were conducted in 25 L glass tanks containing 20 L tap water at 13 and 23 °C, respectively. Both GBs were tested in three replicates. Water samples for N and P analyses were taken right before the GB addition (0 h) and then at every sixth hour for 24 h. The experiment was repeated at 23 °C with daily sampling for 5 days. In both 24-h and 5-day long experiments, the mean GB mass to water volume ratio was 113.4 ± 3.1 mg L^−1^.

### Outdoor tank experiments—nutrient release from GBs in presence and absence of fish

Conducting a series of outdoor tank experiment, we investigated the nutrient release from unconsumed and consumed GB. Under a canopy net tent, we set twelve cylindrical, aerated outdoor flow-through tanks (height 60 cm, diameter 80 cm) and filled with 250 L water from the nearby oxbow lake (of River Körös, Szarvas city). Based on the GB type (i.e. No-GB, FM-GB or PB-GB) and the presence/absence of fish, altogether five treatment types were applied: (1) FM-GB × No-fish and (2) PB-GB × No-fish represented the effect of unconsumed GBs; (3) FM-GB × Fish and (4) PB-GB × Fish represented the effect of consumed GB; and (5) No-GB × Fish represented the basic endogenous (i.e. not feeding-related) nutrient excretion of fish. Because we had only twelve tanks, experimental trials were organized according to a balanced incomplete block design (BIBD; Fig. S1). Four of the five treatment types in three replicates were tested in each of the five trials and tested treatments were rotated between trials as that: each treatment type represented in four trials; and each pair of treatment types represented in identical number (three) of trials.

Tanks with fish were populated with three 1 year old common carp (*Cyprinus carpio*) 7 days before the first trial providing acclimatisation. The mean total fish biomass per tank was 1133 ± 143 g. Common carp is one of the most preferred game fish in Europe^14,54^ and the size-group (300–500 g) used in our experiment is frequently stocked promoting recreational fishery (e.g.^55,56^). Experimental fish originated from nearby small ponds filled with the same oxbow lake water that was used in the experiment.

The five, 24-h long trials were conducted between 16 May and 11 June and at increasing mean water temperatures of 14 °C, 17 °C, 20 °C, 22 °C and 26 °C, respectively. Water temperature followed the natural temperature regime of the oxbow lake. At the beginning of each trial, water flow was closed, reference water samples were taken, and GB was added. Applied amount of GB was 2% of the fish biomass in tanks with fish, and the average of the former tanks in fishless tanks. Post-treatment water samples were taken 24 h later and then the water flow was opened again. Between the trials, 5 to 7 days resting periods were provided for fish. Both during the pre-experiment acclimatisation and the between trials resting periods, fish were fed with formulated aquafeeds, but 1 day starvation applied before each trial. Experiments was accordance with permit for use of animals for scientific purposes (permit number: BE/25/4302-3/2017, issued by Department of Food-security and Animal Health, Békés County).

Temperature and dissolved oxygen were measured at least twice a day during the whole study. No fish mortality occurred during the study, but two fish jumped out of the thank during the first resting period, and thus, were replaced with healthy and similar sized fish.

### Sample analysis—N and P measurements

Nutrient (i.e. forms of N and P) content and concentration analysis of GBs and water samples were carried out in the accredited laboratory of Hungarian University of Agriculture and Life Sciences (Research Center for Irrigation and Water Management, Laboratory for Environmental Analytics).

Groundbait samples were dried to constant mass at 60 °C and homogenized with mortar and pestle. Kjeldahl method was applied for N content analysis. Phosphorus content of GBs was determined using microwave-assisted nitric acid-hydrogen peroxide digestion and subsequent ICP-OES (iCAP 6500 Duo View, Thermo Scientific) measurement.

Water samples were immediately analysed for ammonia, nitrite, nitrate, and orthophosphate using automatic flow injection analysis system (Quikchem 8500, Lachat Instruments). Potassium persulfate digestion method was used for total N (TN) and total P (TP) determination; liberated N and P forms were measured in automatic analysis unit (Ganimede-N and P, Hach Lange).

### Statistical analysis

In laboratory experiments on nutrient dissolution from GBs, the amount of dissolved total N and P was calculated as the difference between the measured total N and P content of the tank water at the observational and the reference time points (i.e. just before the GB was added), and expressed in percentage of the initial nutrient content of GB. Data were evaluated with mixed-design analysis of variance (ANOVA): GB type and temperature (only in the 24-h long experiment) were between-subject factors and time was a within-subject (repeated measures) factor. One significant outlier score was found and removed from the analysis in both N and P data of the 5-days long experiment, at day five. Assumption of (approximate) normality was checked by visual inspection of Q–Q plot of residuals, and assumption of sphericity was checked by using the Mauchly’s test. Tukey post-hoc test with Bonferroni adjusted p-criteria was used to evaluate pairwise differences between treatments at each observational time point and between time points for each treatment type.

In outdoor experiments on nutrient release from GBs in presence and absence of fish, we evaluated treatment related changes (i.e. difference between pre-treatment and end of the trial measurements) of concentrations of N and P forms (i.e. inorganic, organic and total) in the tank water. Data were evaluated first with general linear model (GLM) analysis considering trials (i.e. blocks in BIBD data structure) as random effect factor and treatment as fixed effect factor. Assumptions of the GLM analysis were checked at the same way as in the laboratory experiments, and preliminary analysis proved that fish (ANOVA: trial, F_4,29_ = 0.55, p = 0.700; treatment, F_2,29_ = 0.04, p = 0.965) and GB mass (ANOVA: trial, F_4,40_ = 0.96, p = 0.438; treatment, F_3,40_ = 0.30, p = 0.828) were homogeneous across trials and treatment types where participated. Data of each trial were then analysed separately for between treatment differences with one-way ANOVA followed by Bonferroni adjusted Tukey post-hoc test. Finally, since water temperature varied considerably between trials, and some of the underlying processes of the experiment could be temperature dependent, data of each treatment type were also analysed separately for temperature related (corresponding also to between trials) differences with one-way ANOVA followed by Bonferroni adjusted Tukey post-hoc test.

### Supplementary Information

Supplementary Figure S1.

## Data Availability

The datasets generated during the study are available from the corresponding author on reasonable request.
