# Supplementary material for: Acute effects of angler’s groundbaits: nutrient flux to water column
Source: Sci Rep. 2023 Oct 17;13:17691. doi: 10.1038/s41598-023-44381-3 (PMC10582090; doi:10.1038/s41598-023-44381-3)
Supplement: Supplementary file 1 — Supplementary Figure S1. [file 41598_2023_44381_MOESM1_ESM.docx]

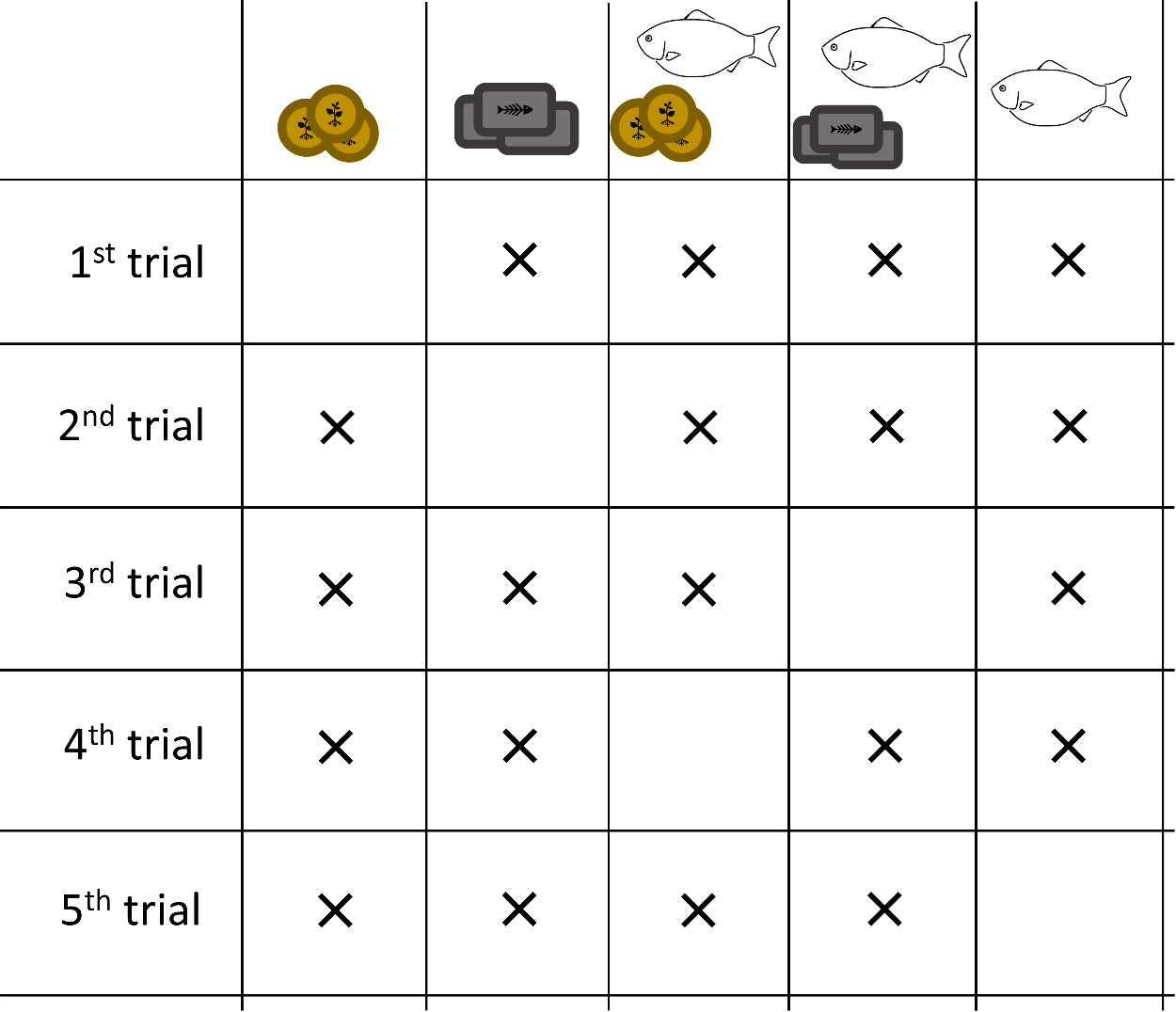


Fig. S1. Design (balanced incomplete block) of outdoor tank experiments. Each treatment type within a given trial was run in triplicate.
